# Autism spectrum disorder: Cadmium and mercury concentrations in different biological samples, a systematic literature review and meta-analysis of human studies

**DOI:** 10.1016/j.heliyon.2024.e27789

**Published:** 2024-03-08

**Authors:** Zana Ramazani, Samaneh Nakhaee, Kiomars Sharafi, Zaynab Rezaei, Borhan Mansouri

**Affiliations:** a Substance Abuse Prevention Research Center, Research Institute for Health, Kermanshah University of Medical Sciences, Kermanshah, Iran; b Medical Toxicology and Drug Abuse Research Center (MTDRC), Birjand University of Medical Sciences, Birjand, Iran; c Research Center for Environmental Determinants of Health (RCEDH), Research Institute for Health, Kermanshah University of Medical Sciences, Kermanshah, Iran

**Keywords:** Cadmium, Mercury, Autism, Heavy metal, Children

## Abstract

The present study was conducted to investigate the differences in cadmium (Cd) and mercury (Hg) concentrations between children with autism spectrum disorder (ASD) and controls. In this systematic review and meta-analysis study, three thousand one hundred forty-five studies were collected from scientific databases including Web of Science, Scopus, PubMed, and Google Scholar from January 2000 to October 2022 and were investigated for eligibility. As a result, 37 studies published in the period from 2003 to 2022 met our inclusion criteria and were considered in the meta-analysis. The heterogeneity assumption was evaluated using the Chi-squared-based Q-test and I-squared (I^2^) statistics. The pooled estimates were shown in the forest plots with Hedges’ g (95% confidence interval) values. The random effects model demonstrated that there is no significant difference in the blood (Hedges' g: 0.14, 95% CI: 0.45, 0.72, *p* > 0.05), hair (Hedges' g: 0.12, 95% CI: 0.26, 0.50, *p* > 0.05), and urinary (Hedges' g: 0.05, 95% CI: 0.86, 0.76, *p* > 0.05) Cd levels of the case group versus control subjects. Moreover, the pooled findings of studies showed no significant difference in the blood (Hedges' g: 1.69, 95% CI: 0.09, 3.48, *p* > 0.05), hair (Hedges' g: 3.42, 95% CI: 1.96, 8.80, *p* > 0.05), and urinary (Hedges' g: 0.49, 95% CI: 1.29 – 0.30, *p* > 0.05) Hg concentrations. The results demonstrated no significant differences in Hg and Cd concentrations in different biological samples of children with ASD compared to control subjects.

## 1 Introduction

Autism Spectrum Disorder (ASD) is a complex neurodevelopmental disorder that affects multiple cognitive functions. It is characterized by persistent problems in verbal and non-verbal social interactions, social skills, memory functions, flexibility abilities, and repetitive patterns of behavior [[1], [2], [3], [4]]. ASD was first characterized in 1943 and since then, there has been a significant increase in its incidence worldwide [3]. It is worth noting that ASD is almost always associated with psychiatric disorders and thus leads to serious health problems [1]. It not only affects the children but can also impose burdens on supporting families, caregivers, and health care services [1]. Currently, there is no known cure for ASD, and children with ASD require ongoing and consistent treatment and care [5]. Medical management of ASD has mainly relied on medication, but research has shown that non-pharmacological treatments, specifically behavioral therapies, are the most effective way to address the core symptoms of ASD [6]. Conventional non-medication treatments, such as behavioral interventions, speech, and language therapy, are used for children of all ages and are provided in various settings [7]. Additionally, some emerging treatments have been previously produced, including dietary changes, nutritional supplements, and non-biological treatments. Some promising therapies include antioxidants, melatonin, acetylcholinesterase inhibitors, naltrexone, and music therapy [5]. To date, the etiology of this disorder has not been elucidated [3]. However, previous research has led to the identification of ASD as a heterogeneous disorder that may result from a combination of different factors such as genetic, epigenetic, and environmental agents [1]. ASD is likely caused by interactions between multiple genes, as well as variations in gene expression due to epigenetic factors and exposure to environmental factors [8].

Environmental factors associated with ASD include complications during pregnancy and childbirth, viral infections, autoimmune diseases, and exposure to substances that can cause birth defects [8]. Several neurotoxic environmental factors have been recognized that may cause neurodevelopmental disability and ASD [3]. However, there are still many debates about the effects of environmental factors after birth [9,10]. Toxic metals are among the possible environmental factors that may play a role in the development of ASD during pregnancy or after birth [1]. Heavy metals are not biodegradable and can easily accumulate in the tissue [[11], [12], [13]], may have the potential to cause serious complications in most organs and increase the likelihood of developing diseases [1,14,15]. Excessive exposures to heavy metals have harmful effects on the nervous system. The developing nervous system of children is especially more vulnerable to heavy metal toxicity compared to adults [[16], [17], [18]]. Also, the risk rises as a result of the cumulative and additive effect of metals. Heavy metals impair the function of multiple enzymes, disrupt processes of cell signaling, and cause oxidative stress that leads to apoptosis [3]. The cumulative impact of harmful environmental damage can result in oxidative stress and damage to neurons in susceptible genetically individuals [5].

Among toxic heavy metals, high levels of cadmium (Cd) and mercury (Hg) are most commonly observed in children with ASD [3]. Various reports on Cd concentrations in children with ASD are presented. Some documents have reported elevated levels of Cd in children with ASD compared to healthy ones [[19], [20], [21]], while other studies have shown no significant association between Cd levels and ASD occurrence [[22], [23], [24]]. Of the numerous literatures that have assessed the associations between Hg exposure and ASD, most studies have shown that Hg could be considered a risk factor for ASD [19,20,25]. However, some studies reported contradictory results [21,22]. So, it is not easy to evaluate the totality of the evidence [2]. Therefore, this systematic review and meta-analysis study was conducted to compare the concentration of Cd and Hg in different biological samples of children with ASD and control subjects using the results from published studies.

## 2 Materials and methods

**Fig. 1:**
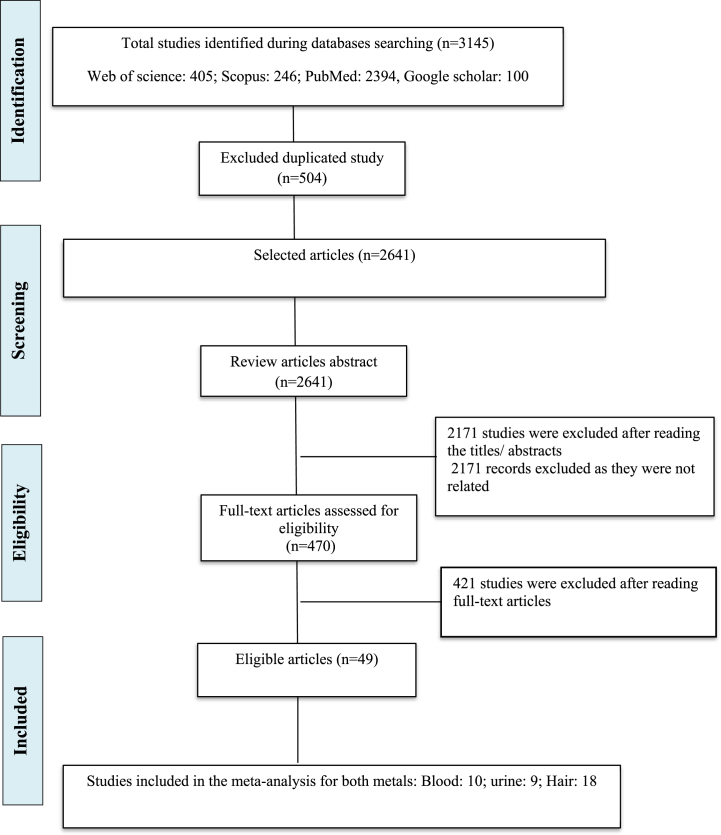
PRISMA flow diagram for **the** identification, inclusion, and exclusion of studies.

According to the PRISMA flowchart, all the required information extracted from the articles was collected and used for both systematic review and meta-analysis sections (Fig. 1).

### 2.1 Information sources and search strategy

In this section, two researchers separately searched relevant articles for eligible studies through the most important databases such as Web of Science, PubMed, Scopus, and Google Scholar. Keywords were selected based on the Mesh Terms format and past articles, and the period was determined from January 2000 to October 2022 to find related observational articles. The pattern of finding related articles in the scientific databases was as follows: Scopus and Google Scholar: (TITLE-ABS-KEY (“Autism”) OR TITLE-ABS-KEY (“Autism spectrum disorder”) AND TITLE-ABS-KEY (“trace element”) OR TITLE-ABS-KEY (“toxic metal”) OR TITLE-ABS-KEY (“non-essential element”) OR TITLE-ABS-KEY (“toxic heavy metal”) OR TITLE-ABS-KEY (“cadmium”) OR TITLE-ABS-KEY (“mercury”) AND TITLE-ABS-KEY (“urine”) OR TITLE-ABS-KEY (“urinary”) OR TITLE-ABS-KEY (“Blood”) OR TITLE-ABS-KEY (“hair”) AND TITLE-ABS-KEY (“child”) OR TITLE-ABS-KEY (“children")); Web of Science: TS=(Autism OR Autism spectrum disorder) AND TS=(toxic metal OR toxic heavy metal OR non-essential element OR trace element OR cadmium OR mercury) AND TS=(urine OR Blood OR hair) AND TS=(child OR children); and PubMed: “Autism spectrum disorder" [Mesh]) AND “Child" [Mesh]) AND “Metals, Heavy" [Mesh])" OR Trace Elements" [Mesh]) OR “Poisoning" [Mesh]) OR “toxicity” [Subheading]) OR “cadmium" [Mesh] OR “mercury" [Mesh]) AND “Blood" [Mesh]) OR “Urine" [Mesh]) OR “Hair" [Mesh]. To avoid missing information, we tried to identify additional publications by searching the references used in related studies or through the authors’ awareness of published studies.

### 2.2 Selection criteria

In this study, documents on two groups of children with ASD and healthy subjects were considered. Letters to the editor, conference papers, review articles, and meta-analyses were also deleted from the publication list. The language searched in the databases was English.

### 2.3 Data extraction

The documents were evaluated by two researchers to check the inclusion and exclusion criteria of the articles. In the following, duplicate and unrelated articles in different databases were separated and removed from the research process after reading the titles and abstracts. After this stage, all articles were read in full text, so articles that did not meet the criteria defined in the eligibility criteria were excluded. If there was a difference of opinion between the two researchers about the study inclusion/exclusion of an article, it was evaluated by a third researcher, and the problem was resolved. The most basic parts of the obtained articles are presented in Table 1, which includes information such as the author's name, country of study, studied population, sample size, type of studied metal, type of biological sample, mean age of participants, diagnostic criteria for ASD, and the main results.

**Table 1:** The most important information extracted from the studies for both **the** systematic review and meta-analysis sections.

Authors/year	Study population	Sample size (number)	Metal	Tissue	Mean age (y)	The diagnostic criterion for ASD	Mean ± SD levels of Hg/Cd	Outcome	Final score^a^	Country
Chehbani et al., /2020 [22]	ASD and neurotypical child	ASD: 89 NeurotypicalChild: 70	Cd/Hg	Blood	Case: 7.52 Control: 7.81	DSM-IV	Hg: ASD: 0.86 ± 1.24 Control: 0.77 ± 0.53 Cd: ASD: 0.08 ± 0.13 Control: 0.07 ± 0.13	No significant difference between the two groups	6	Tunisia
Metwally et al. (2015) [19]	ASD and neurotypical child	ASD: 55 (16 female and 39 male) Neurotypical child: 75 (18 female and 57 male)	Cd/Hg	Blood	Case: 4.01 Control: 4.02	DSM-V	Hg: ASD: 11.03 ± 6.63 Control: 2.22 ± 0.55 Cd: ASD: 1.81 ± 2.61 Control: 1.32 ± 0.54	Cd and Hg levels were higher in the ASD group compared to their controls.	7	Egypt
Laura et al., 2011 [41]	ASD and neurotypical child	ASD: 28 (7 female and 21 male) Neurotypical child: 32 (12 female and 20 male)	Cd	Blood	–	DSM	Cd: ASD: 1.29 ± 0.42 Control: 0.94 ± 0.38	A significant difference in Cd between both groups	6	Italy
Rahbar et al. (2021) [23]	ASD and neurotypical child	ASD: 30 Neurotypical child: 30	Cd/Hg	Blood	2 to 12	DSM-IV-TR, CARS, VABS	–	No significant difference between the two groups	5	Pakistan
Qin et al. (2018) [20]	ASD and neurotypical child	ASD: 34 (14 female and 20 male) cNormal child: 38 (17 female and 21 male)	Cd/Hg	Blood	Case: 4.10 ± 0.81 (male) and 4.28 ± 1.06 (female) Control: 4.29 ± 1.73 (male) and 4.35 ± 1.99 (female)	DSM IV	Hg: ASD: 3.89 ± 0.82 Control: 1.13 ± 1.05 Cd: ASD: 0.67 ± 0.29 Control: 0.27 ± 0.23	A significant difference between the two groups	7	China
Hertz-Picco et al. (2010) [42]	ASD and neurotypical child	ASD: 249 Neurotypical child: 143	Cd/Hg	Blood	Case: 2-5 Control: 2-5	ADOS, ADI-R, MSEL, VABS	Hg: ASD: 0.49 ± 1.08 Control: 0.6 ± 1.03 Cd: ASD: 0.15 ± 0.09 Control: 0.23 ± 0.35	No significant difference between the two groups	6	USA
Albiaazti et al. (2012) [43]	ASD and neurotypical child	ASD: 17 (2 female and 15 male) Neurotypical child: 20 (5 female and 15 male)	Cd/Hg	Hair, Urine, Blood	Case: 11.52 Control: 10.41	DSM-IV, ADOS	Hg in blood: ASD: 0.67 ± 0.31 Control: 0.57 ± 0.34 Hg in hair: ASD: 0.32 ± 0.04 Control: 0.28 ± 0.08 Hg in urine: ASD: 0.69 ± 0.07 Control: 0.7 ± 0.07 Cd in urine: ASD: 0.08 ± 0.02 Control: 0.07 ± 0.02 Cd in hair: ASD: 0.07 ± 0.04 Control: 0.08 ± 0.03	No significant difference between the two groups	7	Italy
Li et al. (2018) [44]	ASD and neurotypical child	ASD: 180 (30 female and 150 male) Neurotypical child: 184 (38 male and 146 female)	Cd/Hg	Blood/hair/	Case: 5.06 ± 1.37 Control: 6.12 ± 1.69	DSM IV	Hg in blood: ASD: 55.59 ± 52.56 Control: 13.47 ± 17.24 Hg in hair: ASD: 55.59 ± 52.56 Control: 13.47 ± 17.24 Cd in blood: ASD: 0.25 ± 0.24 Control:0.52 ± 0.26	Hg in was significant in both groups, while Cd was not significant	7	China
Zhao et al. (2022) [21]	ASD and neurotypical child	ASD: 30 (21 male and 9 female) Neurotypical child: 30 (15 male and 15 female)	Cd/Hg	Blood/Urine	Case: 4.2 ± 1.5 Control: 3.8 ± 1.3	CARS**	Hg in blood: ASD: 0.792 ± 0.4818 Control: 0.7556 ± 0.5013 Hg in urine: ASD: 0.1742 ± 0.1954 Control: 0.1413 ± 0.179 Cd in blood: ASD: 0.1929 ± 0.1448 Control:0.1198 ± 0.0685 Cd in urine: ASD: 0.1302 ± 0.1409 Control: 0.0904 ± 0.0919	Cd was significant in both groups, while Hg was not significant	7	China
Adams et al. (2013) [24]	ASD and neurotypical child	ASD: 55 (6 female and 49 male) Neurotypical child: 44 (5 female and 39 male)	Cd	Blood/Urine	Case: 10.00 Control: 11.00	–	Cd in blood: ASD: 0.64 ± 0.23 Control: 0.79 ± 0.23 Cd in urine: ASD: 30.8 ± 90 Control: 17.9 ± 23.6	No significant difference between the two groups	5	USA
Al-Ayadhi (2005) [45]	ASD and neurotypical child	ASD: 65 Neurotypical child: 80	Cd/Hg	Hair	Case: 9.0 Control: 7.2	Clinical judgment, Criteria E−2	Hg: ASD: 4.2 ± 9.1 Control: 0.71 ± 2.03 Cd: ASD: 0.08 ± 0.003 Control: 0.003 ± 0.001	Hg was significant in both groups, while Cd was not significant	6	Saudi Arabia
Aljumaili et al. (2021) [46]	ASD and neurotypical child	ASD: 75 Neurotypical child: 25	Cd/Hg	Hair	Case: 3-14 Control: 3-14	DSM-V	Hg: ASD: 0.216 ± 0.187 Control: 0.092 ± 0.081 Cd: ASD: 0.547 ± 0.157 Control: 0.387 ± 0.156	A statistically significant difference in both groups	6	Iraq
Al-Farsi et al. (2013) [47]	ASD and neurotypical child	ASD: 27 Neurotypical child: 27	Cd	Hair	Case: 9.0 Control: 7.2	Clinical judgment, Criteria E−2	–	A significant difference between the two groups	5	Oman
Blaurock-Busch et al. (2011) [48]	ASD and neurotypical child	ASD: 25 (3 female and 22 male) Neurotypical child: 25 (6 female and 19 male)	Cd	Hair	Case: 6.24 Control: 6.80	DSM-IV, ABC	Cd: ASD: 0.62 ± 0.19 Control: 0.32 ± 0.5	No significant difference between the two groups	6	Saudi Arabia
Fido and Al-Saad (2005) [49]	ASD and neurotypical child	ASD: 40 Neurotypical child: 40	Cd/Hg	Hair	Case: 4.2 ± 2.2 Control: 4.3 ± 2.6	DSM-IV-R	Hg: ASD: 4.5 ± 0.61 Control: 0.31 ± 0.12 Cd: ASD: 0.14 ± 0.03 Control: 0.15 ± 0.03	Hg was significant between both groups, while Cd was not significant	6	Kuwait
De Palma et al. (2012) [50]	ASD and neurotypical child	ASD: 44 Neurotypical child: 61	Cd/Hg	Hair	Case: 9.0 ± 4.0 Control: 8.4 ± 3.1	DSM IV CARS	Hg: ASD: 0.23 ± 0.22 Control: 0.2 ± 0.15 Cd: ASD: 0.0157 ± 0.0184 Control: 0.0172 ± 0.0301	No significant difference between the two groups	7	Italy
Filon et al. (2017) [51]	ASD and neurotypical child	ASD: 30 (5 female and 25 male) Neurotypical child: 30 (5 female and 25 male)	Cd	Hair	Case: 5.2 ± 1.5 Control: 5.0 ± 1.5	–	Cd: ASD: 0.224 ± 0.114 Control:0.063 ± 0.028	A statistically significant difference between the two groups	5	Poland
Tinkov et al. (2019) [52]	ASD and neurotypical child	ASD: 30 Neurotypical child: 30	Cd	Hair	Case: 4.7 ± 1.8 Control: 4.8 ± 2.2	CARS CGI-S***	Cd: ASD: 0.022 ± 0.011 Control: 0.022 ± 0.02	No significant difference between the two groups	5	Russia
Long et al. (2019) [53]	ASD and neurotypical child	ASD: 75 Neurotypical child: 135	Cd	Hair	–	DPCR- ICD-10	–	No significant difference between the two groups	5	Denmark
Skalny et al. (2017a) [54]	ASD and neurotypical child	ASD: 74 Neurotypical child: 74	Cd/Hg	Hair	Case: 5.12 ± 2.3 Control: 5.11 ± 2.3	–	Hg: ASD: 0.1429 ± 0.152 Control: 0.2012 ± 0.2094 Cd: ASD: 0.024 ± 0.015 Control:0.034 ± 0.033	No significant difference between the two groups	5	Russia
Skalny et al. (2017b) [39]	ASD and neurotypical child	ASD: 33 Neurotypical child: 33	Cd/Hg	Hair	Case: 5.0 ± 1.7 Control: 5.0 ± 1.7	DSM-IV-TR	Hg: ASD: 0.1294 ± 0.1114 Control: 0.2308 ± 0.2772 Cd: ASD: 0.038 ± 0.031 Control: 0.033 ± 0.028	No significant difference between the two groups	7	Russia
Adams et al. (2006) [55]	ASD and neurotypical child	ASD: 51 Neurotypical child: 40	Cd/Hg	Hair	Case: 7.1 ± 3.0 Control: 7.5 ± 3.0	Clinical judgment	Hg: ASD: 0.24 ± 0.23 Control: 0.22 ± 0.18 Cd: ASD: 0.069 ± 0.21 Control: 0.124 ± 0.14	No significant difference between the two groups	6	USA
Kern et al. (2007) [56]	ASD and neurotypical child	ASD: 45 (10 female and 35 male) Neurotypical child: 45 (10 female and 35 male)	Cd/Hg	Hair	Case: 3.0 Control: 3.0	DSM-IV, clinical judgment	Hg: ASD: 0.14 ± 0.11 Control: 0.16 ± 0.1 Cd: ASD: 0.58 ± 0.45 Control: 0.82 ± 0.64	Cd was significant between both groups, while Hg was not significant	6	USA
Fiore et al. (2020) [57]	ASD and neurotypical child	ASD: 48 (34 male and 14 female)	Cd/Hg	Hair	6.5 ± 3.8 y	DSM-5	–	Cd was significant between both groups, while Hg was not significant	4	Italy
Yorbik et al. (2010) [58]	ASD and neurotypical child	ASD: 30 (6 female and 24 male) Neurotypical child: 20 (7 female and 13 male)	Cd	Urine	Case: 6.9 Control: 5.6	DSM-IV, ABC	Cd: ASD: 0.45 ± 0.32 Control:1.43 ± 1.16	No significant difference between the two groups	7	Turkey
Domingues et al., /2016 [59]	ASD and neurotypical child	ASD: 19 (4 female and 15 male) Neurotypical child: 21 (4 female and 17 male)	Cd/Hg	Urine	Case: 6.9 Control: 0.7.4	DSM IV-TR, ADOS	Hg: ASD: 0.55 ± 0.13 Control: 0.74 ± 0.27 Cd: ASD: 0.02 ± 0.01 Control: 0.04 ± 0.01	No significant difference between the two groups	6	Italy
Blazewicz et al. (2022) [60]	ASD and neurotypical child	ASD: 129 (108 male and 21 female) Neurotypical child: 86 (54 male and 32 female)	Cd	Urine	Case: 14.1 ± 1.4 Control: 14.7 ± 1.2	ADOS-2, ADI-R	Cd: ASD: 0.499 ± 0.359 Control: 0.127 ± 0.163	A significant difference between the two groups	7	Poland
Rezaei et al. (2022) [61]	ASD and neurotypical child	ASD: 44 (21 male and 9 female) Neurotypical child: 35 (15 male and 15 female)	Cd/Hg	Urine	Case: 11.1 ± 2.2 Control: 10.4 ± 2.9	DSM-IV	Cd: ASD: 0.28 ± 0.14 Control: 0.22 ± 0.14	A significant difference between the two groups	6	Iran
Yassa et al., 2014 [62]	ASD and neurotypical child	ASD: 45 (13 female and 32 male) Neurotypical child: 45 (13 female and 32 male)	Hg	Blood/Hair	Case: 4.01 Control: 4.02	DSM-V	Hg in blood: ASD: 4.02 ± 0. Control: 0.01 ± 0.02 Hg in hair: ASD: 5.21 ± 0.0 Control: 0.11 ± 0.02	A significant difference between the two groups	7	Egypt
Ip et al. (2004) [63]	ASD and neurotypical child	ASD: 82 (9 female and 73 male) Neurotypical child: 55 (9 girls and 46 boys)	Hg	Blood/Hair	Case: 7.2 ± 0.2 Control: 7.8 ± 0.4	DSM-IV	Hg in hair: ASD: 2.26 ± 0.2 Control: 2.07 ± 0.58 Hg in blood: ASD: 19.53 ± 5.56 Control: 17.68 ± 2.48	No significant difference between the two groups	6	Hong Kong
Macedoni-Lukšič et al. (2015) [25]	ASD and neurotypical child	ASD: 52 Neurotypical child: 22	Hg	Blood	Case: 6.2 ± 3.0 Control: 6.6 ± 3.7	DSM-V, DSM-IV-TR	Hg: ASD: 1.9 ± 0.97 Control: 1.55 ± 0.56	A significant difference between the two groups	7	Slovenia
Abdel Hack et al. (2020) [64]	ASD and neurotypical child	ASD: 30 (21 male and 9 female) Neurotypical child: 30 (15 male and 15 female)	Hg	Blood	Case: 6.5 ± 2.4 Control: 5.4 ± 1.8	DSM-V AND CARS**	–	A significant difference between the two groups	7	Egypt
El-Baz et al. (2010) [65]	ASD and neurotypical child	ASD: 32 (10 female and 22 male) Neurotypical child: 15 (6 female and 9 male)	Hg	Hair	Case: 6.7 ± 3.2 Control: 5.5 ± 2.7	DSM-IV-TR	Hg: ASD: 0.79 ± 0.51 Control: 0.21 ± 0.08	A significant difference between the two groups	7	Egypt
Elsheshtawy et al. (2011) [66]	ASD and neurotypical child	ASD: 32 (8 female and 24 male) Neurotypical child: 32 (8 female and 24 male)	Hg	Hair	Case: 4.1 Control: 4.0	DSM-IV	Hg: ASD: 0.55 ± 0.06 Control: 3.2 ± 0.2	A significant difference between the two groups	7	Egypt
Mohamed et al. (2015) [67]	ASD and neurotypical child	ASD: 100 (16 female and 84 male) Neurotypical child: 100 (26 female and 74 male)	Hg	Hair	Case: 6.2 ± 4.2 Control: 6.8	DSM-IV-TR	Hg: ASD: 0.39 ± 0.37 Control: 0.25 ± 0.16	A significant difference between the two groups	7	Egypt
El-Ansary et al. (2017) [68]	ASD and neurotypical child	ASD: 35 Neurotypical child: 30	Hg	Hair	Case: 7.00 Control: 7.2	DSM-IV-TR	Hg: ASD: 1.73 ± 0.4 Control: 1.51 ± 0.53	A significant difference between the two groups	7	Saudi Arab
Hodgson (2014) [69]	ASD and neurotypical child	ASD: 27 Neurotypical child: 272	Hg	Hair	Case: 7.6 ± 1.4 Control: 7.7 ± 1.3	Clinical judgment	Hg: ASD: 6.93 ± 0.36 Control: 0.61 ± 0.03	A significant difference between the two groups	6	South Korea
Priya and Geetha (2011) [70]	ASD and neurotypical child	ASD 15 Neurotypical child: 50	Hg	Hair	Case: 4-12 Control: 4-12	CARS	Hg: ASD: 5.12 ± 0.61 Control: 2.87 ± 0.34	A significant difference between the two groups	6	India
Gil-Hernnsez et al. (2020) [71]	ASD and neurotypical child	ASD: 54 Neurotypical child: 54	Hg	Hair	Case: 3-9 Control: 3-9	DSM-V	Hg: ASD: 8.26 ± 10.57 Control: 13 ± 12.68	A significant difference between the two groups	6	Spain
Majewska et al. (2010) [72]	ASD and neurotypical child	ASD: 91 Neurotypical child: 75	Hg	Hair	Case: 2-6 Control: 2-6	DSM-V, ADO-2, ADI-R, CARS, PDDBI	Hg: ASD: 0.13 ± 0.16 Control: 0.23 ± 0.29	No significant difference between the two groups	6	Poland
Amdas et al. (2008) [73]	ASD and neurotypical child	ASD: 78 Neurotypical child: 31	Hg	Hair	Case: Control:	–	Hg: ASD: 0.87 ± 2.6 Control: 0.95 ± 0.87	No significant difference between the two groups	7	USA
Holmes et al. (2003) [74]	ASD and neurotypical child	ASD: 94 Neurotypical child: 45	Hg	Hair	Case: 17.7 (11–24)**** Control: 17.8 (12–24)	DSM-IV	Hg: ASD: 0.47 ± 0.28 Control: 3.63 ± 3.56	A significant difference between the two groups	7	USA
Williams et al. (2008) [75]	ASD and neurotypical child	ASD: 15 Neurotypical child: 16	Hg	Hair	Case: 6-12 Control: 6-12	DSM-5-TR	Hg: ASD: 0.07 ± 0.06 Control: 0.08 ± 0.07	No significant difference between the two groups	6	USA
Kaiuzan-Czaplinskaet et al. (2012) [76]	ASD and neurotypical child	ASD: 15 Neurotypical child: 16	Hg	Hair	Case: 6-12 Control: 6-12	DSM-5-TR	–	No significant difference between the two groups	6	USA
Gaza et al. (2017) [77]	ASD and neurotypical child	ASD: 20 Neurotypical child: 20	Hg	Hair	5-17 y	Clinical judgment	Hg: ASD: 1.7 ± 0.078 Control: 1.54 ± 0.13	No significant difference between the two groups	5	Indonesia
Yasuda et al. (2005) [78]	ASD and neurotypical child	ASD: 200 Neurotypical child: 56	Hg	Hair	4-9 y	–	Hg: ASD: 3.25 ± 0.41 Control: 3.39 ± 0.48	Hg was significant between both groups, while Hg was not significant	4	Japan
Zhou et al. (2021) [79]	ASD and neurotypical child	ASD: 50 (37 male and 13 female)	Hg	Hair	3.5 ± 1.3 y	DSM-5	–	No significant difference between the two groups	4	China
Waligora et al. (2019) [80]	ASD and neurotypical child	ASD: 20 Neurotypical child: 18	Hg	Hair/Urine	Case: 0-15 Control: 0-15	Clinical judgment	Hg in hair: ASD: 0.261 ± 0.205 Control: 0.207 ± 0.212 Hg in urine: ASD: 0.152 ± 0.091 Control:0.232 ± 0.353	No significant difference between the two groups	6	Poland
Wright et al. (2012) [81]	ASD and neurotypical child	ASD: 56 Neurotypical child: 121	Hg	Urine	Case: 9.6 ± 3.6 Control: 12.6 ± 3.5	RDC, ADI-R, ADOS-G	Hg: ASD: 6.61 ± 6.95 Control: 4.38 ± 4.34	No significant difference between the two groups	7	UK

a Based on the Newcastle-Ottawa Scale for cross-sectional studies; **Childhood Autism Rating Scale (CARS), ***Clinical Global Impression-Severity scale (CGI-S); ****Median months at baby.

### 2.4 Quality assessment

Based on the Newcastle-Ottawa Scale (NOS), the methodological quality of the included studies was evaluated. We then assessed a study that scored five points or more as high quality and this was according to the standards presented in previous publications; otherwise, it was considered low quality [26].

### 2.5 Statistical analysis

In this research, STATA 17.0 software was applied to analyze the data of the meta-analysis section. To indicate pooled estimates, we applied the forest plots with Hedges' g and 95% CIs. When considerable heterogeneity (*I*^*2*^ statistic more than 70% and *p*-value of Q-test <0.1) was detected, the pooled estimates were analyzed using a random-effects model; otherwise, a fixed-effect model was used. On the other hand, using the I-squared test (I^2^) and Chi-square-based Q-test, the heterogeneity of the included studies was evaluated. Publication bias in this research was assessed in two ways, including visual inspection of funnel plots and using Egger's test or the Begg test. Moreover, it was used to calculate the number of possible excluded studies from the trim-and-fill approach.

## 3 Results

### 3.1 Study selection

Searching different databases provided a total of 3145 documents, of which 504 documents were excluded due to duplication. The title/abstract (n = 2134) and full-text (n = 470) of the obtained articles were evaluated (Fig. 1). Finally, 49 documents met the inclusion criteria and were considered for assessment of quality (Table 1).

### 3.2 Study characteristics

Forty-nine included studies in the systematic review evaluated at least one of the two heavy metals (Cd and Hg) in the urine, blood, and hair of children with ASD compared to healthy children. The studies had sufficient data (mean ± SD levels of Hg/Cd in different samples - blood, urine, and hair - in two groups of children with ASD and controls) that were considered in our meta-analysis (n = 37 studies). These documents were published between the years 2003 and 2022. Most of them had a case-control design.

### 3.3 Systematic review

#### 3.3.1 Cadmium

Twenty-eight studies analyzed Cd in various biological samples (hair, blood, and urine) of children with ASD and healthy controls. Most of them had a case-control design. Among these studies, 4, 2, and 7 documents with quality scores of 4–7 demonstrated significant differences in the mean levels of Cd between the two groups in the blood, urine, and hair samples, respectively. The remaining studies (n = 6, 4, 11) with quality scores of 5–7 did not report significant differences in Cd concentrations in the blood, urine, and hair samples, respectively.

#### 3.3.2 Mercury

Forty studies analyzed Hg concentrations in biological samples (hair, blood, and urine) of children with ASD and healthy ones. Most of them had a case-control design. Among these studies, 6, 5, and 18 documents with quality scores of 4–7 confirmed no significant differences in mean Hg concentrations between the groups in the hair, urine, and blood samples. The remaining studies (n = 6, 0, 12) with quality scores of 6–7 documented a significant difference in Hg concentrations in the blood, urine, and hair samples.

### 3.4 Meta-analysis

#### 3.4.1 Blood cadmium levels

**Fig. 2:**
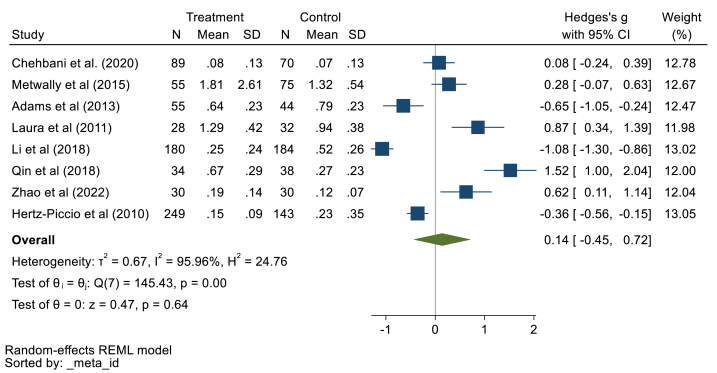
Point and pooled estimates of Hedge's *g* effect size with 95% confidence intervals of Cd concentration in blood samples of children with ASD compared to healthy children. For each primary study, the sample size (n), mean, standard deviation (SD), and Hedge's *g* value with 95% CI are shown. Heterogeneity indices are also presented.

Eight documents with 720 children with ASD and 616 healthy controls were examined. Pooling data using the random effect model demonstrated that there is no significant difference in blood Cd concentrations of children with ASD compared to healthy ones (Hedges' g: 0.14, 95% CI: 0.45, 0.72, p > 0.05), 95% prediction interval: 1.49, 2.27 (Fig. 2). We found heterogeneity among the studies (*I*^*2*^ = 95.96%, τ2 = 0.67, Q = 145.43, *p* < 0.001). The Begg test results (z = 2.10, p = 0.03) presented a publication bias. Sensitivity analyses suggested a good robustness of overall effects (range: 0.05, 0.32) (supplementary file, Fig. 1). Because all studies include the 95% confidence interval (CI) of the overall estimate (range 95% CI: 0.57, 0.88). The meta-regression results showed that age has a significant association (B = −1.33, p = 0.03), but gender had no confounding effect (B = 0.12, p = 0.68).

#### 3.4.2 Hair cadmium levels

**Fig. 3:**
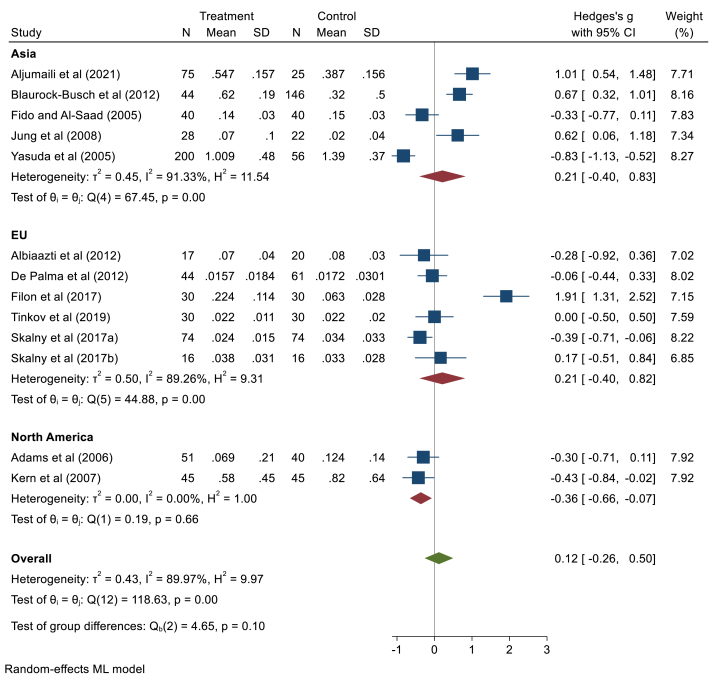
Point and pooled estimates of Hedge's *g* effect size with 95% confidence intervals of Cd concentration in hair samples of children with ASD compared to healthy children, stratified by different continents after excluding outlier studies (Al-Ayadhi (2005) and Gaza (2017)). For each primary study, the sample size (n), mean, standard deviation (SD), and Hedge's *g* value with 95% CI are shown. Heterogeneity indices are also presented.

In this section, 15 studies with 779 children with ASD and 705 children in the healthy group were compared. The random effect model exhibited that there are no significant differences in Cd concentrations of hair in children with ASD compared to controls (Hedges' g: 4.43, 95% CI: 1.19, 10.06, p > 0.05), 95% prediction interval: 21.18, 30.09 (supplementary file, Fig. 2). The studies were heterogeneous (*I*^*2*^ = 99.95, τ2 = 122.27, Q = 486.44, *p* < 0.001). It seems that the Al-Ayadhi (2005) and Gaza (2017) study has a relatively large influence because the 95% CI from the meta-analysis excluding that study, [−1.74, 5.75] and [−2.23,7.35], does not contain the overall effect size estimate based on all studies, 4.4 (supplementary file, Fig. 3). After removing the Al-Ayadhi (2005) and Gaza (2017) study, The random effect model exhibited that there are no significant differences in Cd concentrations of hair in children with ASD compared to controls (Hedges' g: 0.12, 95% CI: 0.26, 0.50, p = 0.56) (Fig. 3). The results of subgroup analysis based on different continents showed that pooled concentrations of hair Cd in Asia (Hedges' g: 0.21, 95% CI: 0.4, 0.83, p > 0.05) and Europe (Hedges' g: 0.21, 95% CI: 0.4, 0.82, p > 0.05) had no significant differences. But the pooled results of two studies from North America showed significant differences (Hedges' g: 0.36, 95% CI: 0.66, −0.07, p < 0.05). Based on the Egger test results (z = 1.86, p = 0.062), no publication bias was observed. The meta-regression results showed that age has no significant association (B = 0.36, p = 0.73), and gender had no confounding effect (B = 0.04, p = 0.71).

#### 3.4.3 Urinary cadmium levels

**Fig. 4:**
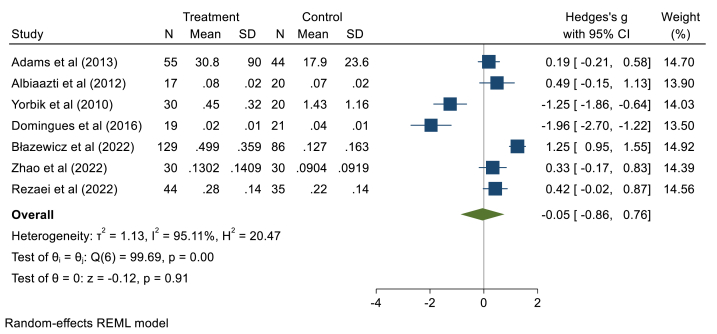
Point and pooled estimates of Hedge's *g* effect size with 95% confidence intervals of Cd concentration in urine samples of children with ASD compared to healthy children. For each primary study, the sample size (n), mean, standard deviation (SD), and Hedge's *g* value with 95% CI are shown. Heterogeneity indices are also presented.

Seven studies with 324 ASD children and 256 healthy children were considered in the meta-analysis. These studies were heterogeneous (*I*^*2*^ = 95.11, τ2 = 1.13, Q = 99.69, *p* < 0.001) so the random effect model was performed. Pooling data of seven studies showed that there are no significant differences between the children with ASD and the healthy subjects in urinary Cd levels (Hedges' g: 0.05, 95% CI: 0.86, 0.76, *p* > 0.05), 95% prediction interval: 2.98, 2.88 (Fig. 4). The Begg test presented a publication bias among included documents (z = −3.08, P = 0.002). Sensitivity analyses suggested a good robustness of overall effects (range: 0.27, 0.26). Because all studies include the 95% confidence interval of the overall estimate (range 95%CI: 1.09, 0.98) (supplementary file, Fig. 4). The meta-regression results showed that age has no significant association (B = 0.41,p = 0.46), and gender had no confounding effect (B = −0.07, p = 0.20).

#### 3.4.4 Blood mercury levels

**Fig. 5:**
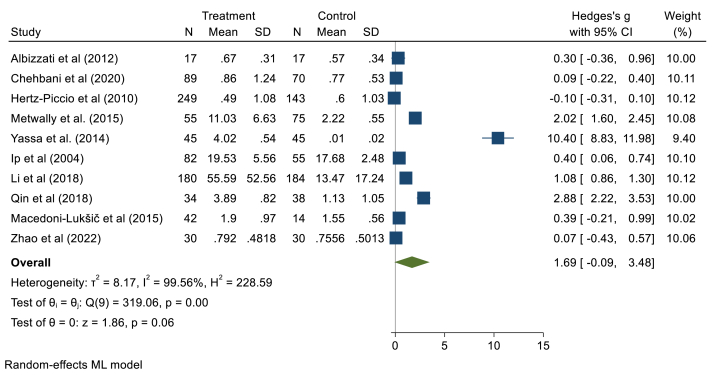
Point and pooled estimates of Hedge's *g* effect size with 95% confidence intervals of Hg concentration in blood samples of children with ASD compared to healthy children. For each primary study, the sample size (n), mean, standard deviation (SD), and Hedge's *g* value with 95% CI are shown. Heterogeneity indices are also presented.

In this section, ten studies with 803 children with ASD and 649 subjects in the control group were included. The random effect model showed that there is no significant difference in blood Hg concentrations of children with ASD compared to healthy ones (Hedges' g: 1.69, 95% CI: 0.09, 3.48, p > 0.05), 95% prediction interval: 5.64, 9.04 (Fig. 5). The pooled studies were heterogeneous (*I*^*2*^ = 99.56, τ2 = 8.17, Q = 319.06, *p* < 0.001). The Begg test showed a publication bias among included documents (z = 5.96, *P* = 0.0001). It seems that the Yassa (2014) study has a relatively large influence because the 95% CI from the meta-analysis excluding that study, [0.13, 1.43], does not contain the overall effect size estimate based on all studies, 1.69 (supplementary file, Fig. 5). The meta-regression results showed that age has no significant association (B = −1.20,p = 0.63), and gender had no confounding effect (B = 0.26,p = 0.21).

#### 3.4.5 Hair mercury levels

**Fig. 6:**
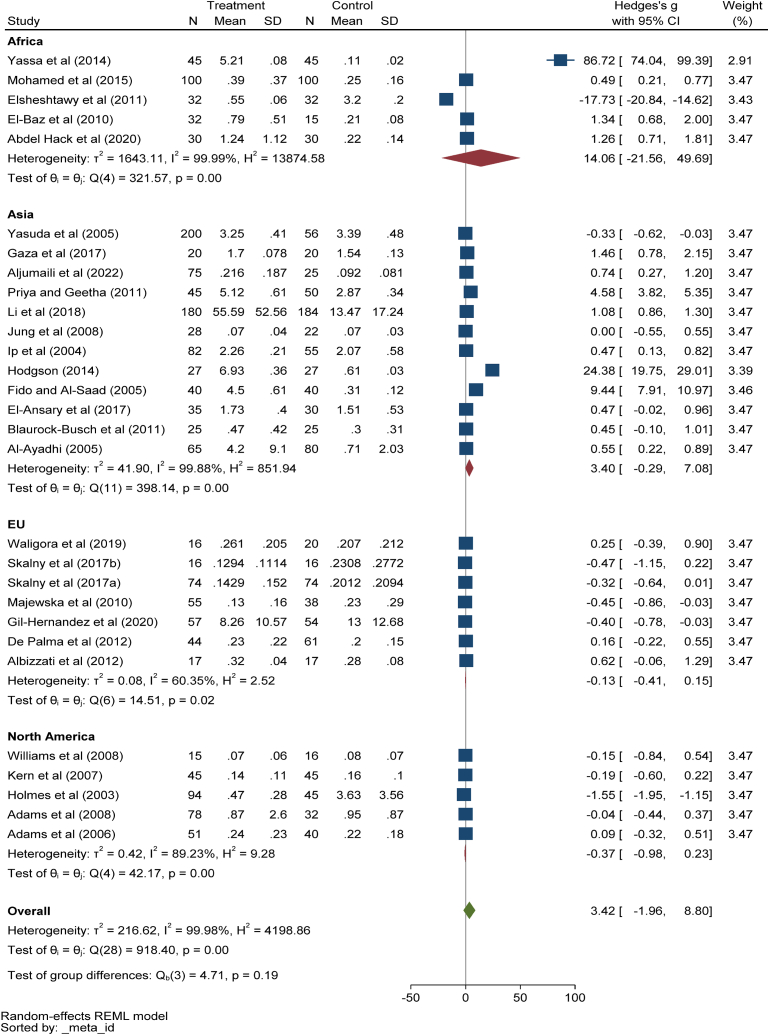
Point and pooled estimates of Hedge's *g* effect size with 95% confidence intervals of Hg concentration in hair samples of children with ASD compared to healthy children, stratified by different continents. For each primary study, the sample size (n), mean, standard deviation (SD), and Hedge's *g* value with 95% CI are shown. Heterogeneity indices are also presented.

Twenty-nine studies with 1543 and 1294 children in ASD and control groups were considered in the meta-analysis. The included documents were heterogeneous (*I*^*2*^ = 99.98%, τ2 = 216.62, Q = 918.4, *p* < 0.001) so, the random effect model was used. Pooling data of 29 studies showed no significant differences between the children with ASD and the healthy ones in hair Hg levels (Hedges' g: 3.42, 95% CI: 1.96, 8.80, *p* > 0.05), 95% prediction interval: 27.29, 34.13 (Fig. 6). The results of subgroup analysis based on different continents showed that pooled concentrations of hair Hg in Africa (Hedges' g: 14.06, 95% CI: 21.56, 49.69, p > 0.05), Asia (Hedges' g: 3.4, 95% CI: 0.29, 7.08, p > 0.05) and Europe (Hedges' g: 0.13, 95% CI: 0.41, 0.15, p > 0.05), and North America (Hedges' g: 0.37, 95% CI: 0.98, 0.23, p > 0.05) had no significant differences (Fig. 6). The Egger test results (z = 9.03, P = 0.0001) demonstrated a publication bias. It seems that the Yassa 2014 study has a relatively large influence because the 95% CI from the meta-analysis excluding that study, [−1.18, 2.88], does not contain the overall effect size estimate based on all studies, 3.4 (supplementary file, Fig. 6). The meta-regression results showed that age has no significant association (B = −3.48,p = 0.78), and gender had no confounding effect (B = 1.09,p = 0.46).

#### 3.4.6 Urinary mercury levels

**Fig. 7:**
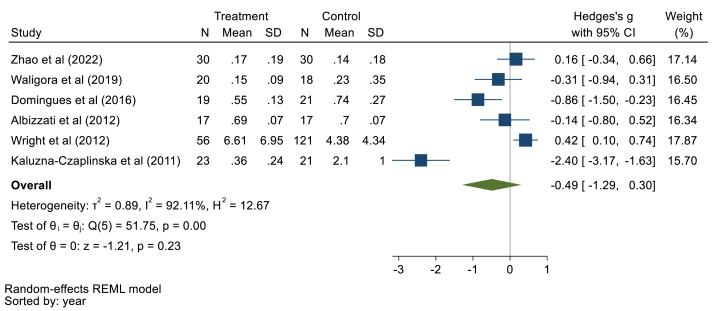
Point and pooled estimates of Hedge's *g* effect size with 95% confidence intervals of Hg concentration in urine samples of children with ASD compared to healthy children. For each primary study, the sample size (n), mean, standard deviation (SD), and Hedge's *g* value with 95% CI are shown. Heterogeneity indices are also presented.

Six studies with 120 children with ASD and 228 healthy children were examined. Pooled data demonstrated no significant differences in urinary Hg concentrations of children with ASD compared to healthy controls using the random effect model (Hedges' g: 0.49, 95% CI: 1.29, 0.30, *p* > 0.05), 95% prediction interval: 3.34, 2.36 (Fig. 7). The included studies had heterogeneity (I^2^ = 92.11 %, τ2 = 0.89, Q = 51.75, *p* < 0.01) with a publication bias (Begg test, z = −2.25, P = 0.06). Sensitivity analyses suggested a good robustness of overall effects (range: 0.57, −0.10) because all studies include the 95% confidence interval of the overall estimate (range 95%CI: 1.56, 0.53) (supplementary file, Fig. 7). The meta-regression results showed that age has no significant association (B = −0.01,p = 0.99), and gender had no confounding effect (B = −0.05, p = 0.98).

## 4 Discussion

Numerous investigations are being conducted worldwide to clarify the contributing factors to ASD development [27]. Various factors have been considered for the pathogenesis of ASD, and the potential role of environmental pollutants, such as toxic metals in ASD development, has been of great interest in scientific communities [27]. This study presented the pooled results of studies focusing on Cd and Hg concentrations. The results demonstrated no significant differences in Cd and Hg concentrations in different biological samples of children with ASD compared to healthy subjects. Different factors should be considered in interpreting the results. There were many variations across the included studies in our meta-analysis, and there were confounding factors that were difficult to account for, such as diet, nutritional status, exposure levels, medications, socioeconomic status, education, maternal age, health status, and exposure to other pollutants/heavy metals. Additionally, the definition of ASD is very broad and includes multiple subtypes, reflecting the heterogeneity in the disease etiology. In different individual studies, total Hg was measured but not in organic or inorganic forms separately. Exposure to organic forms of Hg such as ethyl mercury (used as a preservative in thimerosal vaccine) and methyl mercury (present mainly in seafood products) are believed to be more involved in ASD [28]. Furthermore, the co-exposures or interaction of other trace elements may cause synergistic or antagonistic effects, or the biological deficiencies may exacerbate the toxic metal effects in the human body. For example, the selenium (Se) and Hg ratio may show an antagonistic function against Se concentrations. A significantly lower Se/Hg ratio has been reported in ASD patients [29]. This strengthens the hypothesis that a deficiency of Se increases Hg neurotoxicity in ASD.

Rossignol et al. (2014) conducted a systematic review of case-control studies for five toxic metals (Pb, Cd, Hg, and As) in the blood, urine, hair, tooth, or brain. For all toxic metals except As, more than half of the documents demonstrated no increase in any of the measurements in children with ASD compared with healthy ones. They also reported seven studies that identified associations between heavy metal concentrations (mostly Hg and Pb) and ASD severity [30]. Saghazadeh (2017) in a meta-analysis reported no differences in hair and urinary Cd concentrations between ASD patients and healthy children. They showed that the development status of countries significantly affects hair Cd concentrations. Subgroup analyses revealed that children with ASD in developing regions but not in developed regions had elevated hair Hg levels compared to control subjects [1]. Also, in our study, the subgroup analysis indicated that the pooled data from North America showed significantly lower levels of hair Cd in children with ASD. Additionally, data in Asian countries had wider confidence intervals than European and American countries', relatively narrow confidence intervals indicating more heterogeneity in studies from Asian countries. Stricter protocols and advanced equipment may influence this observation in measuring heavy metal levels in developed nations.

Results of another meta-analysis (2017) showed that Hg concentration in the brain tissue and blood of children with ASD was significantly higher compared to healthy children [28]. They found that three of the eleven investigations that measured Hg in blood revealed a positive relationship with ASD, and the rest reported no significant link with ASD. Pooling all results in the meta-regression showed a positive relationship between Hg and ASD [28]. Yoshimasu et al. (2014) conducted a meta-analysis study assessing the association of early infancy or prenatal exposures with Hg and ASD. They reported that thimerosal vaccine injection (containing ethyl mercury) was not associated with ASD, whereas environmental exposure to Hg was significantly associated with this disorder [31]. It has been shown that urinary levels of some specific biomarkers for assessing Hg exposure, such as precoproporphyrin (prcP), coproporphyrin (cP), and pentacarboxyporphyrin (5cxP), are considered to be related to ASD staging [28,[32], [33], [34], [35]].

The causal effect of Hg exposure on neurodevelopmental diseases is still unclear [36]. It is supposed that Hg exerts its harmful effects mainly by interacting with enzymes and proteins or by generating reactive oxygen species (ROS). Mercury ions can bind to phosphoryl, carboxyl, sulfhydryl, amine, and amide groups in the protein, which causes the inactivation of the Hg-binding proteins [37]. The possible mechanisms of cadmium's effects on the nervous system include disruption of the brain-blood-barrier, interference with cell proliferation and differentiation, brain oxidative stress, interference with DNA repair, and apoptosis which disrupts the balance of neurotransmitters [1,16,27,38].

The conflicting results of review studies may be related to the periods and the methods of analysis in different studies. In relation to studies that showed higher levels of heavy metals in children with ASD compared to healthy ones, it should be considered that children with ASD may present more mouthing behaviors than healthy ones, leading to elevated concentrations of Cd, Hg, and other pollutants in their various biological samples. In the case of no significant differences of toxic metals between autistic children and healthy ones, it does not necessarily mean that there is no association because the metals may have accumulated in the central nervous system [27,39].

Our study has limitations; in this study, we compared the concentrations of each metal separately, and we cannot assess the possible mitigating or potentiating effects of other agents that may influence the exposure consequences. No cohort study could be considered in the meta-analysis. All investigations had a case-control or cross-sectional design and thus they may not provide the effects during a period. Many of the included documents are limited by small sample sizes, and the precise time of exposure to heavy metals in relation to the neurodevelopmental disorder has not been specified in the different studies. Because we only looked at published studies, as in other systematic reviews, there is potential for publication bias. Importantly, the criteria for the diagnosis of ASD have changed over the years and included studies have used different criteria. One factor that we could not consider in this study is the biological differences and effects of disease severity. ASD is more commonly seen in boys than girls and previous documents showed that girls are diagnosed later than boys, suggesting that it is more difficult to diagnose the disease in girls [40]. It is important to clarify whether boys are more exposed to heavy metals than girls.

## 5 Conclusion

The meta-analysis results provided no significant differences in Cd and Hg concentrations in various biological samples (urine, blood, and hair) of children with ASD compared to healthy subjects. The pooled data from North America showed significantly lower levels of hair Cd in children with ASD. Also, hair Cd levels were not significantly different between ASD children and healthy ones on different continents.

## Availability of data and materials

The data used and analyzed for the current study are available from the corresponding author upon request.

## Ethics approval and consent to participate

This study was approved by the Research and Ethics Committee of Kermanshah University of Medical Sciences (IR.KUMS.REC.1399.700).

## Consent for publication

Not applicable.

## Funding

This project was generously supported financially by the 10.13039/501100005317Kermanshah University of Medical Sciences (Grant number: 1399.700).

## CRediT authorship contribution statement

**Zana Ramazani:** Conceptualization, Methodology, Validation, Writing - Original Draft, Writing - Review & Editing. **Samaneh Nakhaee:** Methodology, Data curation, Writing - Original draft preparation, Writing- Review & Editing, Formal analysis, Investigation. **Kiomars Sharafi:** Methodology, Data curation, Writing - Original draft preparation, Review & Editing. **Zaynab Rezaei:** Methodology, Data curation, Writing - Original draft preparation, Review & Editing. **Borhan Mansouri:** Conceptualization, Supervision, Methodology, Validation, Writing - Original Draft, Writing - Review & Editing, Data Curation, Formal analysis.

## Declaration of competing interest

The authors declare that they have no known competing financial interests or personal relationships that could have appeared to influence the work reported in this paper.
