# Supplementary material for: Autism spectrum disorder: Cadmium and mercury concentrations in different biological samples, a systematic literature review and meta-analysis of human studies
Source: Heliyon. 2024 Mar 8;10(6):e27789. doi: 10.1016/j.heliyon.2024.e27789 (PMC10944282; doi:10.1016/j.heliyon.2024.e27789)
Supplement: Multimedia component 1 [file mmc1.docx]

**Fig1:** sensitivity analysis for blood cadmium levels

**Fig 2.** Point and Pooled estimates of Hedge’s g effect size with 95% confidence intervals of cadmium concentration in hair samples of autistic children compared to healthy children stratified by different continents. For each primary study, the sample size (n), mean, standard deviation (SD), and Hedge’s g value with 95% CI are shown. Heterogeneity indices are also presented

**Fig 3:** sensitivity analysis for hair cadmium levels

**Fig 4:** sensitivity analysis for urinary cadmium levels

**Fig 5:** sensitivity analysis for blood mercury levels

**Fig 6:** sensitivity analysis for hair mercury levels

**Fig 7:** sensitivity analysis for urinary mercury levels
